# Simulating droplet motion on virtual leaf surfaces

**DOI:** 10.1098/rsos.140528

**Published:** 2015-05-20

**Authors:** Lisa C. Mayo, Scott W. McCue, Timothy J. Moroney, W. Alison Forster, Daryl M. Kempthorne, John A. Belward, Ian W. Turner

**Affiliations:** 1 Mathematical Sciences, Queensland University of Technology, Brisbane, Queensland 4001, Australia; 2 Plant Protection Chemistry NZ Ltd, Rotorua, New Zealand

**Keywords:** thin film, liquid drop, coalescence, curvilinear, alternating direction implicit methods

## Abstract

A curvilinear thin film model is used to simulate the motion of droplets on a virtual leaf surface, with a view to better understand the retention of agricultural sprays on plants. The governing model, adapted from Roy *et al.* (2002 *J. Fluid Mech.* 454, 235–261 (doi:10.1017/S0022112001007133)) with the addition of a disjoining pressure term, describes the gravity- and curvature-driven flow of a small droplet on a complex substrate: a cotton leaf reconstructed from digitized scan data. Coalescence is the key mechanism behind spray coating of foliage, and our simulations demonstrate that various experimentally observed coalescence behaviours can be reproduced qualitatively. By varying the contact angle over the domain, we also demonstrate that the presence of a chemical defect can act as an obstacle to the droplet's path, causing break-up. In simulations on the virtual leaf, it is found that the movement of a typical spray size droplet is driven almost exclusively by substrate curvature gradients. It is not until droplet mass is sufficiently increased via coalescence that gravity becomes the dominating force.

## 2. Introduction

Droplet motion on leaf surfaces is a complex process, dependent on both the chemical composition of the droplet and the specific leaf surface [1,2]. The topic is of great interest in agrichemical spray applications where the retention of spray droplets needs to be maximized, with an even distribution (coverage) over the leaf canopy of a crop of particular importance for the efficacy of protectant pesticides, which include many fungicides and insecticides. In this context, modelling the interactions that occur between spray drops and leaves is best considered in two parts. First is the impaction event, which may lead to droplet bounce, splash or adhesion [3–8]. Impaction models must quantify the volume of fluid which is captured by the leaf and determine whether any of the repelled fluid is recaptured by another part of the plant. Next, after the successful retention of a drop on a leaf, post-impaction models must adequately describe the drop's journey. Of particular interest is the question of whether the drop interacts with others to coat the surface, or is lost to runoff at the leaf extremities. Depending on the application, further modelling of the leaf uptake of an active ingredient from the fluid formulation may also be required [9].

**Figure 1.**
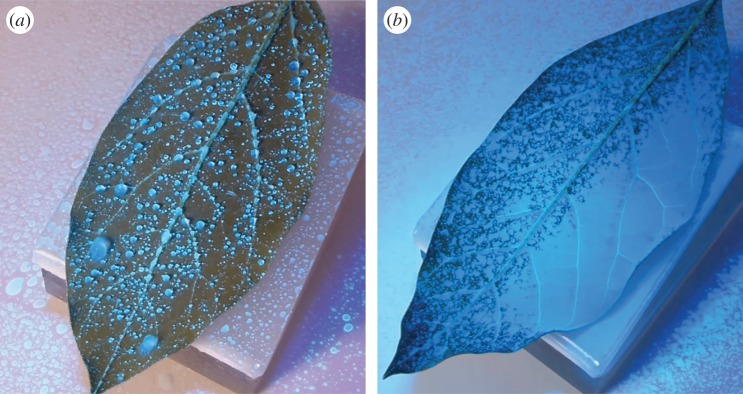
(*a*) The abaxial surface of an avocado leaf is sprayed with water, leading to coalescence and runoff. (*b*) A surfactant (Du-Wett^®^, Etec Crop Solutions Ltd) has been added to the water, causing a coating film to form on the leaf surface. See also the electronic supplementary material.

Experimental observations like those shown in figure 1 provide motivation to further investigate the post-impaction movement of a spray droplet on a leaf surface. In figure 1*a* (and the electronic supplementary material), an accumulation of water sprayed on the difficult-to-wet underside (abaxial surface) of an avocado leaf leads to the coalescence of small stationary droplets into larger ones which then quickly propagate along the leaf surface. As they move, they engulf smaller drops in their path and grow increasingly in size, leaving a clean leaf surface in their wake. These large drops are eventually lost as runoff on the edge of the leaf. In figure 1*b* (and the electronic supplementary material), the captured coalescence behaviour is very different. Here, a surfactant has been added to the spray formulation, lowering the fluid surface tension and consequently the contact angle of the fluid on the leaf (at 1000 ms, the surface tension of the droplet with this particular surfactant at 0.15% concentration is approximately 25 mN m^−1^, as opposed to 72 mN m^−1^ for water). The result is that the individual drops spread on the leaf rather than beading up, leading to a rapid coalescence of neighbouring drops into a thin and even coating film. The spreading observed with the addition of surfactant can be likened to water drops (with no surfactant) spreading on a more easily wettable surface. These interactions are well known for the coalescence and runoff of rain droplets on surfaces of various wettability [10], but are less studied in the context of agricultural sprays on leaves.

We wish to provide simulations of droplet motion which replicate these observations and provide additional insight into the process. The simulations must be capable of incorporating the effects of complex leaf surface topography, as some leaf varieties have significant gradients in elevation and curvature. We employ a thin film model with gravity, substrate curvature and contact angle acting as the influencing forces of droplet motion. The governing model, presented later in §3, is a generalization of the well-studied thin film equation for gravity-driven flow down an inclined plane [11–14]:

2.1 $$\frac{\partial h}{\partial t}=-\frac{1}{3\mu }\nabla \cdot [\gamma h^{3}\nabla \nabla^{2}h-\rho gh^{3}\nabla h\cos\alpha +\rho gh^{3}\sin\alpha \text{i}],$$

where *h*(*x*,*y*,*t*) is the fluid height measured from the plane, *μ* is the fluid viscosity, *γ* is surface tension at the fluid–air interface, *ρ* is density, *g* is acceleration due to gravity and *α* is the inclination angle of the plane. The gradient operator is defined as ∇=(∂_*x*_,∂_*y*_), where *x* is the distance down the incline and *y* in the transverse direction. The main assumptions behind (2.1) are that the film is very thin in comparison to a representative in-plane length scale, that the dimensionless Reynolds number is of O(1) or smaller (for a relatively viscous and slow-moving flow), and that the contact angle of the fluid on the surface is very small (for a complete wetting fluid).

Equation (2.1) describes the time evolution of the free surface of a liquid film within some spatial domain, but it is the movement of the contact line which is often truly of interest (the three-phase interface between the fluid film, the solid substrate and the surrounding gas), as this interface separates the regions of dry domain from wet. However, the existence of a true moving contact line introduces a paradox where the standard no-slip condition on the substrate contradicts the movement that we expect at the contact line [15]. A precursor film model is used to alleviate this problem by assuming that a thin film of dimensionless height *b*≪1 coats the entire domain (where the representative height of the main film is one dimensionless unit). Therefore, there is no longer a true contact line in the model, but an apparent one where the bulk of the fluid in the droplet meets the precursor level. While smaller is better, the choice of *b* is restricted by mesh resolution, as the mesh spacing must be of roughly the same order as *b* or smaller for numerical accuracy [16,17].

Studies of (2.1) are typically concerned with the coating flow of a fluid sheet, often in the context of understanding the stability of an advancing contact line. In the context of flow down a plane, gravity-driven instability manifests as fingering or sawtooth patterns in the front of the film [11–14,17]. With the addition of a disjoining pressure term, equation (2.1) has been shown to also effectively model the flow of fluid droplets, whose movement involves both advancing and receding contact lines. Using (2.1) with a two-term form of the disjoining pressure, Schwartz *et al.* [18] and Koh *et al.* [19] simulated droplets sliding down inclined and vertical planes, demonstrating steady-state, pearling and stretching behaviours depending on plane inclination angle and drop size. While these simulations were qualitatively similar to published experimental findings [20,21], Koh *et al.* [19] noted that quantitative agreement could only be achieved with a finer mesh resolution, and precursor level commensurate with experiments. Koh *et al.* [19], along with Veremieiev *et al.* [22] and Ahmed *et al.* [23] additionally modelled contact angle hysteresis through the prescription of a spatially varying contact angle in the two-term disjoining pressure. On a horizontal surface, Schwartz [24] and Schwartz & Eley [16] demonstrated that a spatially varying contact angle could cause a droplet to spontaneously spread or even split into smaller pieces. The time scale of their simulations was much smaller than observed in reality, again due to the presence of an unrealistically large precursor film. In a similar vein, Schwartz *et al.* [25] modelled surfactant-driven motility and break-up of drops on a horizontal plane.

Other variations of (2.1) incorporate the effect of substrate curvature to model thin film flow on simply curved substrates such as cylinders [26–30] and spheres [31]. Mayo *et al.* [29] simulated droplets sliding down the outer wall of a vertical cylinder, observing the same regimes as those on an inclined plane [18–21]. Other authors have employed a surface profile function to consider the effect of topographical features on droplet movement, such as the channels and microscopic plant surface characteristics presented by Glass *et al.* [32]. Glass *et al.*'s work was driven by the motivation to model the delivery of pesticides to leaf surfaces, and the model also included surfactant and evaporation effects. In a similar vein, Veremieiev *et al.* [22,33] considered the delivery of bio-pesticide to foliage. They approximated a leaf surface with a planar substrate and compared droplet flow on both the top and underside of the substrate. In a study by Gaskell *et al.* [34], droplet motion was influenced by an interplay of contact angle and topographic heterogeneities, demonstrating the possibility of driving preferential spreading patterns. Later, Lee *et al.* [35] simulated the flow of films and drops as obstructed by large occlusions.

Specific to flow on whole leaves is the work of Wang *et al.* [36], in which a ‘virtual surface’ method is used to simulate droplet motion over curved surfaces including leaves. The model considered interfacial tensions at the contact line and implemented a dynamic contact angle, producing realistic droplet motion simulations in the context of animation. Oqielat *et al.* [37] used a triangulated Frangipani leaf surface as the basis for droplet motion. The triangle elements, essentially a series of inclined planes, guided the droplet motion via a path of steepest descent. A crude thin film theory was used to approximate the height of the drop, with motion ceasing when the height decreased below a critical value.

Simple surface topographies have been used to demonstrate the stick–slip motions of two-dimensional droplets [38], which are mostly known to occur on surfaces with chemical heterogeneities (such as a spatially varying contact angle) [39,40]. Savva & Kalliadasis [41] combined both topographic and chemical heterogeneities of the two-dimensional surface to demonstrate these dynamics and showed that chemical gradients could cause a droplet to move uphill. They stated that an ideal homogeneous substrate could not cause droplet pinning, as substrate heterogeneities are the principle source of contact angle hysteresis.

There has clearly been a wealth of research into droplet motion in response to various physical and chemical characteristics, even in the context of movement on leaf-like surfaces. We expect that gradients in the curvature of the leaf surface will play a large role in droplet movement, and while there have been some studies concerned with drop motion over topography, there has been little focus on the effect of substrate curvature. Further, studies of droplet coalescence have mostly focused on sessile droplets [42,43], with motions like those shown in figure 1*a* only well described in the context of rainfall [10]. In this study, we employ a thin film model capable of accurately simulating droplet motion over a virtual leaf surface, which has been reconstructed from three-dimensional scans of a real leaf. We are motivated to recreate observations such as those in figure 1 and to investigate the interplay that occurs between gravity and substrate curvature for a typical spray-size droplet on a leaf.

For flow on complex or arbitrarily curved surfaces, Roy & Schwartz [44], Roy *et al.* [45] and Roberts & Li [46] derived a thin film model in a curvilinear coordinate system. The model accurately describes fluid motion in response to gradients in substrate curvature, as well as gravity and inertia. It is a generalization of (2.1) and will reduce to this form in the case of a planar substrate. The derivation process in [44–46] assumes a special coordinate system, in which lines of principal curvatures are orthogonal (such as on a simply curved substrate like a cylinder or torus). Following Roy *et al.* [45], Thiffeault & Kamhawi [47] presented the curvilinear model in non-orthogonal form, for use when the curved substrate does not have this property. We present the generalized curvilinear model in §3, with the addition of disjoining pressure, to model the gravity- and curvature-driven flow of a small droplet on a virtual leaf substrate.

In §4, a virtual cotton leaf surface, reconstructed from three-dimensional scans of a real leaf, is presented as the substrate for droplet motion. Boundary conditions and numerical details are presented in §5. Our simulations are presented in §6. First, §6.1 investigates the coalescence and separation behaviours of drops on simple surfaces. As shown in figure 1, coalescence is a key mechanism in the interaction between spray droplets on a leaf, and our simulations demonstrate that coalescence behaviour can be reproduced qualitatively. By varying the contact angle over the domain, we also demonstrate the separation of a single drop into multiple parts. This is a representative of leaf surfaces with physical or chemical defects. Finally, §6.2 addresses the problem of droplet motion over a virtual leaf surface. It is found that a typical spray size droplet does not have sufficient mass for gravity to be a key influencing factor in its movement, and instead gradients in substrate curvature drive the flow. However, gravity becomes the dominating force when droplet mass is sufficiently increased. We demonstrate that the coalescence of many spray droplets can lead to the formation of droplets with a larger mass, which may then be driven by gravity.

## 3. Thin film model

Roy & Schwartz [44], Roy *et al.* [45] and then Roberts & Li [46] proposed a model for thin film flow on an arbitrarily curved substrate. This was formed under the assumption of an orthogonal curvilinear coordinate system.

On a leaf surface described by the function *Z*=*f*(*x*^1^,*x*^2^), the curvilinear system is a natural extension of a regular Cartesian system. By superimposing a Cartesian grid on the leaf surface, vectors tangent to the curves of constant *x*^1^ and *x*^2^ (**e**_1_ and **e**_2_, respectively), coupled with normal vectors to the surface (**e**_3_), define a unique basis at any point. On a complexly curved substrate (as opposed to a cylinder, sphere or torus, for example), these bases do not usually align with principal curvatures, and so are not orthogonal. In this case, the orthogonal curvilinear model presented by Roy & Schwartz [44], Roy *et al.* [45] and Roberts & Li [46] becomes slightly more involved.

Thiffeault & Kamhawi [47] have presented the generalized (non-orthogonal) form of Roy *et al.*'s [45] curvilinear model. In order to apply this formulation to our problem, we must first introduce some notation and parameters necessary for the distinction of co- and contravariant objects required for the construction of a curvilinear model. First, note that Greek indices *α* and *β* span the labels 1,2, and that the presence of repeated indices indicates summation, unless otherwise stated. Lower indices denote covariant components, and upper indices contravariant components. By the range convention for index notation, it is assumed that $A_{\alpha \beta }$ denotes all four components of the rank 2 tensor **A** (provided that summation of individual components has not been implied). Partial differentiation with respect to *x*^1^ is represented by ∂_1_, and similarly for ∂_2_.

We first define

3.1 $$p=\partial_{1}Z,\;q=\partial_{2}Z,\;r=\partial_{11}Z,\;s=\partial_{12}Z\;\mathrm{and}\;t=\partial_{22}Z.$$

At a given point on the substrate, the non-orthogonal tangent vectors are

3.2 $${\text{e}}_{1}={\left(1,0,p\right)}^{T}\;\mathrm{and}\;{\text{e}}_{2}={\left(0,1,q\right)}^{T},$$

and the unit normal, given by their cross product, is

3.3 $${\hat{\text{e}}}_{3}=\frac{1}{\sqrt{1+p^{2}+q^{2}}}{\left(-p,-q,1\right)}^{T}.$$

Together these form our basis. The (covariant) metric tensor of the substrate is [47]

3.4 $$G_{\alpha \beta }={\text{e}}_{\alpha }\cdot {\text{e}}_{\beta }=\left[\begin{array}{cc} 1+p^{2} & pq\\ pq & 1+q^{2} \end{array}\right],$$

while its inverse (the contravariant metric tensor) is

3.5 $$G^{\alpha \beta }={\text{e}}^{\alpha }\cdot {\text{e}}^{\beta }=\frac{1}{w^{2}}\left[\begin{array}{cc} 1+q^{2} & -pq\\ -pq & 1+p^{2} \end{array}\right].$$

Here, **e**^*α*^ are the covectors of the substrate (see [47] for full details), and for convenience we define

3.6 $$w=\sqrt{\det\;G_{\alpha \beta }}=\sqrt{1+p^{2}+q^{2}}.$$

The metric tensor and its inverse can be used to raise and lower indices in the usual way, and will be used to express the governing model in terms of the curvilinear coordinates of the substrate. The curvature tensor of the substrate is

3.7 $${K_{\alpha }}^{\beta }=\frac{1}{w^{3}}\left[\begin{array}{cc} (1+q^{2})r-pqs & (1+p^{2})s-pqr\\ (1+q^{2})s-pqt & (1+p^{2})t-pqs \end{array}\right],$$

which gives mean curvature

3.8 $$\kappa ={K_{\alpha }}^{\alpha }=\frac{1}{w^{3}}((1+q^{2})r-2pqs+(1+p^{2})t)$$

and Gaussian curvature

3.9 $$G=\det\;{K_{\alpha }}^{\beta }=\frac{1}{w^{4}}(rt-s^{2}).$$

In the special orthogonal coordinate system assumed in [44–46], the metric and curvature tensors are diagonal. This is not the case for our substrate presented in §4.

The governing thin film equation is derived from the Navier–Stokes equations under lubrication theory (see [44–47] for full details). It is assumed that the fluid film is very thin in relation to its extent across the domain and that the flow occurs predominantly in the *x*^1^ and *x*^2^ directions. A small slope approximation is used for the free surface of the film. The governing equation is to be solved for the film height above the substrate, which is measured at a distance of *y*=*h*(*x*^1^,*x*^2^,*t*) along the normal **e**_3_. As **e**_3_ varies along the surface, it is assumed that the fluid film is sufficiently thin that the normals do not intersect within the thin layer of the fluid. That is, the topographical variations of the substrate are on a sufficiently larger scale than the film thickness.

In coordinate-free form, Thiffeault & Kamhawi's [47] dimensional model is

3.10 $$\begin{array}{rl} \partial_{t}\zeta & =-\frac{\gamma }{3\mu }\nabla \cdot \left[h^{3}\nabla \hat{\kappa }-h^{4}\left(\kappa I-\frac{1}{2}{K_{\alpha }}^{\beta }\right)\nabla \kappa \right]\\ & \;-\frac{\rho g}{3\mu }\nabla \cdot \left[h^{3}\left(I-h\left(\kappa I-\frac{1}{2}{K_{\alpha }}^{\beta }\right)\right){\hat{\text{g}}}_{t}+{\hat{g}}_{n}h^{3}\nabla h\right]-\nabla \cdot [h^{3}\nabla \Pi ] \end{array}$$

and is identical to that presented by Roy & Schwartz [44], Roy *et al.* [45] and Roberts & Li [46] (apart from the disjoining pressure term *Π*, which we have added). The quantity $\zeta =h-\frac{1}{2}\kappa h^{2}+\frac{1}{3}Gh^{3}$ represents the volume per unit substrate area of a fluid layer of thickness *h*, while $\hat{\text{g}}={\hat{\text{g}}}_{t}+{\hat{g}}_{n}{\text{e}}_{3}$ is the gravitational force vector containing tangential and normal components. Further, $\hat{\kappa }=\kappa +\kappa_{2}h+\nabla^{2}h,$ where $\kappa_{2}={K_{\alpha }}^{\beta }{K_{\beta }}^{\alpha }$, and the disjoining pressure *Π* is

3.11 $$\Pi =\frac{\gamma }{Hb}\frac{\left(n-1)(m-1\right)}{\left(n-m\right)}(1-\cos\theta_{e})\left[{\left(\frac{Hb}{h}\right)}^{n}-{\left(\frac{Hb}{h}\right)}^{m}\right].$$

The parameter *H* is the representative height scale, *b* is the dimensionless precursor film height (hence *Hb* is the dimensional precursor height), *θ*_e_ is the equilibrium contact angle of the fluid, and *n* and m are constants such that *n*>m>1. Recall from §2 that the dimensionless precursor film height *b* must be chosen such that *b*≪1. In this study, we use *b*=0.01 (1% of the initial dimensionless drop height and radius).

The introduction of disjoining pressure into the model is necessary to accurately model the contact line movement of a droplet, particularly in the receding portion. This is because the complete wetting assumption of the governing thin film model prevents dewetting of the substrate. The prescription of a contact angle in (3.11) allows the fluid to be partially wetting, aiding movement at the rear of the droplet. While there are many forms of disjoining pressure used in the literature, we choose the above two-term form for its ease of implementation and its compatibility with a precursor film regularization. The first term in square brackets in (3.11) represents liquid–solid repulsion, while the second describes attractive forces. A stable film thickness exists at *h*=*Hb* (the dimensional precursor height). The values of *n* and m are generally chosen to best reflect the physical properties of the system which is being replicated. Here, we use m=2 and *n*=3, a common choice for simulations of droplet movement [16,18,19,24,32].

The disjoining pressure (3.11) results from forces present on an intermolecular scale. Further, it is linked with the precursor thickness which is suggested by experiments to realistically lie within the range of 1 to 100 nm [19,24]. As the numerical scheme must operate on a significantly larger scale, the usage of the disjoining pressure in the current context can be interpreted as a physically motivated device to prescribe an equilibrium contact angle and also to allow de-pinning of the receding contact line [17]. The two-term form of disjoining pressure has been used effectively in many previous studies to model droplet movement down a plane [18,19,22,23], on simply curved surfaces like a cylinder [29], and on more complex surfaces including surface topography [32,35].

By expressing the differential operators of equation (3.11) in the curvilinear coordinates of the substrate, the governing PDE becomes

3.12 $$\partial_{t}\zeta =-\nabla_{\alpha }J^{\alpha },$$

where the covariant divergence is defined as ∇_*α*_*q*^*α*^=(1/*w*)∂_*α*_(*wJ*^*α*^), and the contravariant components of the mass flux vector are

3.13 $$\begin{array}{rl} J^{\alpha }(h) & =\frac{\gamma }{3\mu }h^{3}\left(\partial^{\alpha }\hat{\kappa }-h\kappa \partial^{\alpha }\kappa +\frac{1}{2}h{K_{\beta }}^{\alpha }\partial^{\beta }\kappa \right)\\ & \;+\frac{\rho g}{3\mu }h^{3}\left[{\hat{g}}_{t}^{\alpha }-{\hat{g}}_{t}^{\beta }h\left(\kappa \delta_{\beta }^{\alpha }+\frac{1}{2}{K_{\beta }}^{\alpha }\right)+{\hat{g}}_{n}\partial^{\alpha }h\right]+h^{3}\partial^{\alpha }\Pi . \end{array}$$

The covariant Laplacian (contained within $\hat{\kappa }$) is defined as ∇^2^*h*=(1/*w*)∂_*α*_(∂^*α*^*h*) and $\partial^{\alpha }=G^{\alpha \beta }\partial_{\beta }$. From Thiffeault & Kamhawi [47], the gravitational components are

3.14 $$\hat{\text{g}}={\left({\hat{g}}_{t}^{1},{\hat{g}}_{t}^{2},{\hat{g}}_{n}\right)}^{T}=-\frac{1}{w^{2}}{\left(p,q,w\right)}^{T}.$$

The disparity between height and length scales of the film can be exploited to scale the problem appropriately. The height scale *H* is chosen as the height of the droplet in its equilibrium state on a horizontal plane, while the length scale *R* is the radius. Consequently, *R* is the typical length over which the film height varies by an O(1) magnitude. Applying lubrication theory, it is assumed that *ϵ*=*H*/*R*≪1. The variables are scaled as

3.15 $$\hat{h}=\frac{h}{H},\;(\hat{x},\hat{y})=\frac{\left(x,y\right)}{R},\;{\hat{K}}_{\alpha^{\beta }}=R{K_{\alpha }}^{\beta }\;\mathrm{and}\;\hat{t}=\frac{t}{T},$$

where the time scale *T* is defined as 3*μR*^4^/(*γH*^3^). The metric tensor does not require scaling as it is already dimensionless. Non-dimensionalization of equations (3.1) and (3.11) leads to

3.16 $$\partial_{t}h=-\nabla_{\alpha }J^{\alpha },$$

where

3.17 $$\begin{array}{rl} J^{\alpha }(h) & =h^{3}\left[\partial^{\alpha }\left(\frac{1}{\epsilon }\kappa +\kappa_{2}h+\frac{1}{w}\partial_{\alpha }(\partial^{\alpha }h)\right)-h\kappa \partial^{\alpha }\kappa +\frac{1}{2}h{K_{\beta }}^{\alpha }\partial^{\beta }\kappa \right]\\ & \;+h^{3}\left(\frac{\mathrm{Bo}}{\epsilon }{\hat{g}}_{t}^{\alpha }+\mathrm{Bo}{\hat{g}}_{n}\partial^{\alpha }h\right)+h^{3}\partial^{\alpha }\Pi . \end{array}$$

The new parameter Bo=*ρgR*^2^/*γ* is the Bond number, and the dimensionless disjoining pressure is

3.18 $$\Pi =\frac{2}{b\epsilon^{2}}(1-\cos\theta_{e})\left[{\left(\frac{b}{h}\right)}^{3}-{\left(\frac{b}{h}\right)}^{2}\right].$$

Equation (3.16) is represented to O(1); all terms of O(ϵ) after scaling have been omitted. Note that we have omitted the ‘hat’ notation for readability and will continue to do so from here on.

The relative size of each of the terms in (3.16) can vary greatly with leaf surface topography and droplet properties. For example, if substrate curvature is zero or constant, the curvature terms will not contribute at all to the flow (with zero curvature across the domain, (3.16) reduces to the equation for purely gravity-driven flow down a plane described by (2.1)). If substrate curvature gradients are present, and the droplet's mass is sufficiently small, then ∂^*α*^*κ*/*ϵ* may potentially have the leading order effect on the flow. For a droplet of large mass due to increased volume or density, the tangential gravity terms $\mathrm{Bo}\;{\hat{g}}_{t}^{\alpha }/\epsilon$ may instead dominate the flow, even when substrate curvature gradients are present. The magnitude of the Bond number is of O(1) or smaller in all following simulations.

## 4. Leaf surface fitting

The virtual leaf surface is obtained by digitizing a cotton leaf and reconstructing it with surface fitting techniques. Kempthorne *et al.* [48] discuss three-dimensional scanning devices suitable for the digitization of plant leaves. Here, the Artec S [49] was used to digitize the physical leaf surface, as this device produces the highest quality data upon which to base the surface fitting.

**Figure 2.**
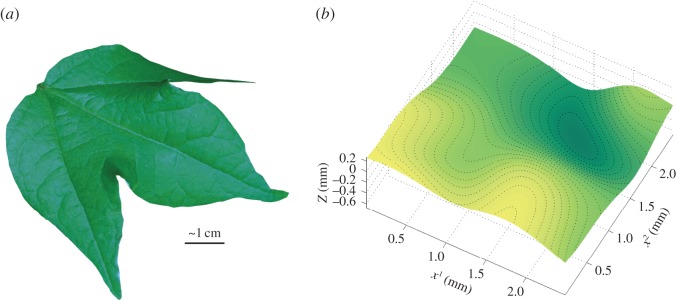
(*a*) The virtual cotton leaf, constructed using a discrete smoothing *D*^2^-spline. A photograph of the cotton leaf has been texture-mapped to the surface. (*b*) For this study, we have chosen a very small, approximately 2.5 mm by 2.5 mm, subset of the leaf surface on which to perform our simulations.

The virtual leaf surface can be reconstructed from the digitized dataset using discrete smoothing *D*^2^-splines [50], as described in Kempthorne *et al.* [51,52]. In [51], the discrete smoothing *D*^2^-spline representing the reconstruction of the full leaf surface was formed as a linear combination of reduced Hsieh–Clough–Tocher (HCT) finite elements, which produce a continuously differentiable (class *C*^1^) surface. This method applied to our leaf surface is shown in figure 2*a*.

The full leaf surface is required for the construction of structural plant models for spray droplet simulations [5], which form the basis of the broader motivation for the present research. In this context, HCT basis functions are favourable due to their compact support. However, they do not produce a class *C*^2^ or higher surface, as required by the parameters of (3.16), which involve at least second-order derivatives of the surface. To overcome this difficulty, polyharmonic splines of the form *ϕ*(*r*)=*r*^5^ were used to reconstruct the small segment of the leaf surface upon which the numerical experiments will take place. This allows the leaf surface to be expressed over this region as

4.1 $$Z(\text{x})=\sum_{i=1}^{n}c_{i}∥\text{x}-{\text{v}}_{i}{∥}^{5},$$

where **v**_*i*_, *i*=1,…,*n* are the centres and the coefficients *c*_*i*_ are to be determined. Figure 2*b* shows a small subset of the leaf surface which has been constructed in this way. This leaf section will be used as the basis of our simulations.

## 5. Numerical details

### 5.1 Boundary and initial conditions

The virtual cotton leaf pictured in figure 2*a* is roughly 8 cm long and 6 cm wide. In this study, a typical spray-sized droplet is assumed to have a spherical diameter of 0.3 mm. This corresponds approximately to the VMD (volume mean diameter) of a medium spray coarseness as per the American Society of Agricultural and Biological Engineers Standard 572.1 [53]. VMD is defined such that 50% of the volume of the spray consists of drops with a smaller diameter, and 50% with a larger. Therefore, a medium spray consists mostly of drops with a diameter *smaller* than 0.3 mm. A 0.3 mm diameter translates to a radius of roughly 0.5 mm when the drop is sitting at equilibrium on the leaf surface with a contact angle of 50°. As a result, a simulated droplet is very small in relation to the leaf surface. Although coalescence and runoff can occur on the whole-leaf scale (figure 1), the following simulations only consider droplet movement over a small rectangular subsection of the leaf surface, as this is sufficient to capture the desired behaviours, and a larger domain can become computationally prohibitive.

As long as the simulated droplet is contained within the boundaries of the set rectangular domain, the particular boundary conditions are not important. We employ simple Dirichlet and Neumann conditions around the perimeter:

5.1 $$\left.\begin{array}{rl} h(x_{a}^{1},x^{2}) & =h(x_{b}^{1},x^{2})=h(x^{1},x_{a}^{2})=h(x^{1},x_{b}^{2})=b\\ \;\text{and}\;\partial_{1}h(x_{a}^{1},x^{2}) & =\partial_{1}h(x_{b}^{1},x^{2})=\partial_{2}h(x^{1},x_{a}^{2})=\partial_{2}h(x^{1},x_{b}^{2})=0, \end{array}\right\}$$

where $x_{a}^{1},x_{b}^{1},x_{a}^{2}$ and $x_{b}^{2}$ denote the *x*^1^ and *x*^2^ boundaries of the domain and *b* is the dimensionless precursor thickness of 0.01.

The initial shape of a droplet is given by

5.2 $$h(x^{1},x^{2},0)=h_{0}\left[1-{\left(\frac{x^{1}-x_{0}^{1}}{r_{0}}\right)}^{2}-{\left(\frac{x^{2}-x_{0}^{2}}{r_{0}}\right)}^{2}\right],$$

where *h*_0_ is the dimensionless height, *r*_0_ the dimensionless radius and $\left(x_{0}^{1},x_{0}^{2}\right)$ the coordinates of the droplet centre. As the length and height scales are the equilibrium radius and height of the drop, a choice of *h*_0_>*r*_0_ would result in a drop spreading to regain its equilibrium, while a drop with *h*_0_<*r*_0_ would contract. Unless otherwise stated, we choose *h*_0_=*r*_0_=1.

### 5.2 Alternating direction implicit scheme

The numerical solution of (3.16) is performed using a pseudolinear alternating direction implicit (ADI) method. Specifically, we employ the pseudolinear scheme presented by Witelski & Bowen [54] as *pL*2, which uses a second-order backward differentiation formula to approximate the time derivative and factorizes (3.16) into the following (coordinate-free) form:

5.3 $$\begin{array}{rl} {\tilde{\text{L}}}_{1}u & =-\frac{1}{3}({\tilde{h}}^{n+1}-4h^{n}+h^{n-1})-\frac{2}{3}\Delta t\nabla \cdot \text{J}({\tilde{h}}^{n+1}), \end{array}$$

5.4 $$\begin{array}{rl} {\tilde{\text{L}}}_{2}v & =u \end{array}$$

5.5 $$\begin{array}{rl} \;\text{and}\;h^{n+1} & ={\tilde{h}}^{n+1}+v, \end{array}$$

where

5.6 $$\text{J}=J^{\alpha }{\text{e}}_{\alpha }={\left(J^{1},J^{2}\right)}^{T}$$

is the flux vector with components given by (3.17), *h*^*n*^ indicates the solution at current time *t*^*n*^, and *h*^*n*±1^ at time *t*^*n*^±Δ*t*, where Δ*t* is the time step. Equations (5.3)–(5.5) are solved in sequence at each time step in order to update the solution from *h*^*n*^ to *h*^*n*+1^. In the general form of *pL*2, this is an iterative process, where the initial iterate is given by the second-order explicit two-level extrapolation ${\tilde{h}}_{\left(0\right)}^{n+1}=2h^{n}-h^{n-1}$, and the iteration generates a sequence of improved estimates ${\tilde{h}}_{\left(k\right)}^{n+1}$ that converge to *h*^*n*+1^. We found that iterating equations (5.3)–(5.5) at each time step provided no measurable improvement to the solution compared with using the method non-iteratively (with a single iteration).

We have discretized **L**_1_ and **L**_2_ using a vertex-centred finite volume scheme (details in appendix A). The linear operators are defined by

5.7 $${\tilde{\text{L}}}_{1}=1+\frac{2}{3}\frac{\Delta t}{A_{P}}\left[\Delta x_{e}^{2}{\left({\tilde{\text{D}}}_{1}\right)}_{e}-\Delta x_{w}^{2}{\left({\tilde{\text{D}}}_{1}\right)}_{w}\right]$$

and

5.8 $${\tilde{\text{L}}}_{2}=1+\frac{2}{3}\frac{\Delta t}{A_{P}}\left[\Delta x_{n}^{1}\;{\left({\tilde{\text{D}}}_{2}\right)}_{n}-\Delta x_{s}^{1}{\left({\tilde{\text{D}}}_{2}\right)}_{s}\right],$$

where e, w, n, s subscripts indicate evaluation at the east, west, north and south control volume (CV) faces, *A*_*P*_ is the surface area of the curved substrate within a given CV, and $\Delta x_{e}^{2}$ and its variations are the length of each control volume face. The differential operators are

5.9 $$\begin{array}{rl} {\tilde{\text{D}}}_{\alpha } & ={\left({\tilde{h}}^{n+1}\right)}^{3}\left\{\left[\kappa_{2}G^{\alpha \alpha }+\mathrm{Bo}{\hat{g}}_{n}G^{\alpha \alpha }+\partial^{\alpha }\left(\frac{1}{w}\partial_{\beta }(wG^{\beta \alpha })\right)\right]\partial_{\alpha }\right.\\ & \;\left.+\left[G^{\alpha \alpha }\left(\frac{1}{w}\partial_{\beta }(wG^{\beta \alpha })\right)+\partial^{\alpha }G^{\alpha \alpha }\right]\partial_{\alpha \alpha }+{\left(G^{\alpha \alpha }\right)}^{2}\partial_{\alpha \alpha \alpha }\right\}\\ & \;+\frac{2}{\epsilon^{2}b}(1-\cos\theta_{e})G^{\alpha \alpha }\left[2b^{2}-3b^{3}{\left({\tilde{h}}^{n+1}\right)}^{-1}\right]\partial_{\alpha }. \end{array}$$

In this case, repeated *α* does not indicate summation, except for the term $\partial_{\beta }(wG^{\beta \alpha })$, for which there is summation over *β*.

The ${\tilde{\text{D}}}_{\alpha }$ operators are obtained by splitting the spatial operators into distinct *x*^1^ and *x*^2^ parts. As a result, equations (5.3)–(5.5) sequentially solve a family of one-dimensional problems by way of *x*^1^- and *x*^2^-direction sweeps of the discretized domain. The use of second-order gradient approximations leads to linear pentadiagonal systems to be solved in each iteration of equations (5.3) and (5.4). An advantage of employing a finite volume discretization is its superior mass conservation properties compared with finite difference schemes.

While ADI methods have been popular in solving thin-film-type equations like ours [18,24,29], more recent work has shown that parallelized multigrid methods may offer the greatest potential for efficiency [55,56]. However, we find that an ADI approach is sufficiently fast for the scale of the presented simulations.

### 5.3 Choice of parameters

Given a droplet volume (such as *V* =0.014 μl for a spray drop of 0.3 mm diameter), we make the assumption that the equilibrium drop formation is a paraboloid with radius *R*, height *H* and volume *V* =*πR*^2^*H*/2. The small slope approximation, as required by the thin film model, gives an estimation of the equilibrium contact angle as *θ*_e_=2*H*/*R*. While the contact angle does not in practice determine the aspect ratio of a drop, this approach allows a method for stipulating reasonable values of *R* and *H* when a drop volume and contact angle are given as inputs.

The prescribed contact angle value can significantly alter the motion of the drop due to the coefficient of the disjoining pressure term (3.11). However, the choice of *θ*_e_ must abide by the constraints of the thin film model. First, a contact angle larger than 90° would imply that *h*(*x*^1^,*x*^2^,*t*) is multivalued, which is not allowed. Second, a choice of *θ*_e_ too close to 90° would result in *H*≈*L*, which contradicts the lubrication assumption that *ϵ*=*H*/*L*≪1. This introduces the first major restriction to our model, as many plant species have very hydrophobic surfaces, leading to large *θ*_e_ for water drops. However, the addition of surfactants to the water acts to significantly reduce this hydrophobicity, and so our model is still valid in such regimes. A small contact angle means that our simulated droplets move via a sliding motion, rather than the rolling motion which is observed for drops with a more circular cross section [57].

In this study, we use values up to *θ*_e_=50°, which results in *ϵ*=0.44. This is not a particularly small value for the thin film parameter, but the model is robust as evidenced by our simulations. Each of the following simulations was performed with a mesh spacing of Δ*x*^1^=Δ*x*^2^=0.01. This was found to be sufficiently small for grid independence of the solution and to provide accurate solutions with a precursor level of *b*=0.01.

Throughout this study, simulated droplets have the density and viscosity properties of water: *ρ*=1000 kg m^−3^ and *μ*=8.9×10^−4^ Pa s, respectively. Surface tension is either specified as *γ*=0.072 N m^−1^ for pure water droplets or *γ*=0.025 N m^−1^ for water with surfactant added. The Bond numbers in the following simulations range from Bo=0.0085 to 2.35, satisfying the requirement that it is of O(1) or smaller.

## 6. Simulations

Previous studies of droplet motion have reproduced steady and transient sliding drop behaviours [18,19], demonstrated preferential droplet motion due to chemical and physical properties of the substrate [16,34], and simulated droplets moving over leaf-like or virtual leaf surfaces [32,36,37]. We wish to further investigate the interaction that occurs between a drop and a curved leaf surface in the context of agricultural sprays, and demonstrate that observations such as those in figure 1 can be reproduced qualitatively with a thin film model. In §6.1, we consider a simple planar substrate in order to focus on the processes of droplet coalescence and separation. In §6.2, we use a reconstructed leaf surface, digitized from a real cotton leaf, to investigate the relative roles that gravity and substrate curvature play in spray droplet motion.

### 6.1 Coalescing and separating drops

**Figure 3.**
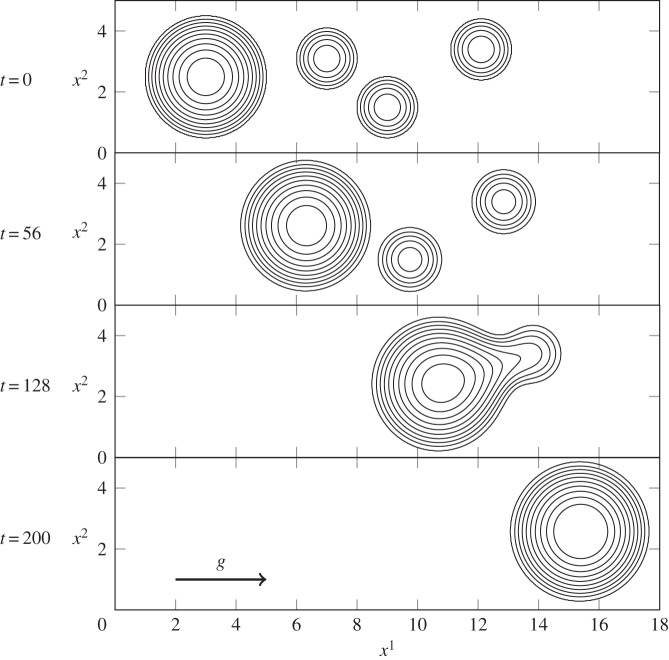
As several drops slide down a vertical plane, the larger drop consumes smaller ones in its path. Parameter values are *γ*=0.072 N m^−1^, *ϵ*=0.44 and Bo=0.034, contour spacing is Δ*h*=0.2.

**Figure 4.**
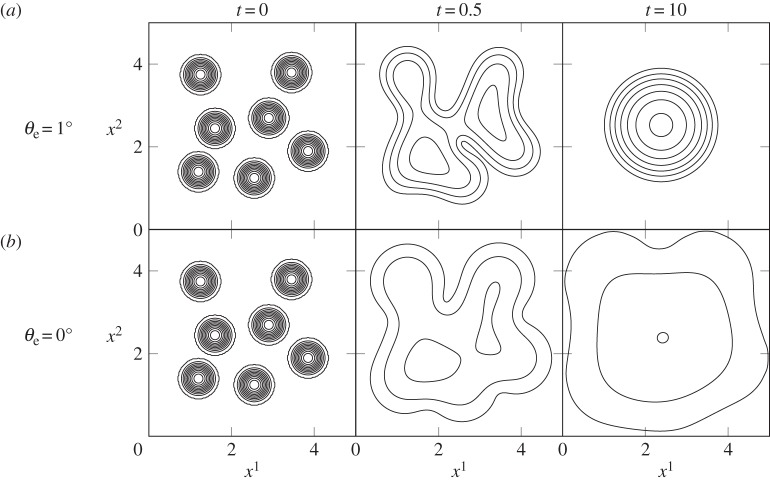
Drops on a horizontal plane spread and coalesce. A single larger drop is formed for a contact angle of *θ*_e_=1° (*a*), while a continuously spreading film of fluid develops for *θ*_e_=0° (*b*). Parameter values for both simulations are *γ*=0.025 N m^−1^, *ϵ*=0.0087 and Bo=0.33, contour spacing is Δ*h*=0.2.

Recall figure 1, in which two different spray formulations lead to contrasting behaviours when delivered to the underside of an avocado leaf. In figure 1*a*, water droplets have a large equilibrium contact angle on this hydrophobic surface, resulting in almost spherical droplets. Coalescence only occurs when two or more drops are sprayed in proximity to each other, or when a drop slides into another. However, the addition of a surfactant in figure 1*b* considerably lowers the surface tension of the spray formulation, and consequently the contact angle. This causes the drops to spread outwards and coalesce rapidly over the leaf surface, leading to a coating film and almost full leaf coverage. In figures 3 and 4, we present simulations of these types of coalescence events.

First, figure 3 depicts a simulation of multiple droplets on a vertical plane. A planar substrate avoids any potentially confounding substrate curvature effects from the analysis, reducing equation (3.16) to the (dimensionless) form of (2.1). The initial condition (*t*=0) for this simulation sees four droplets placed on a vertical wall: a larger towards *x*^1^=0 (corresponding to the top of the plane) and three smaller randomly placed down-plane. While the contact angle for the experiments in figure 1*a* is greater than 90°, the model assumptions require that we prescribe a smaller value. As such, we have chosen to use *θ*_e_=50° here.

As simulation time begins, the drops begin to travel down the wall. The larger and more massive drop travels the fastest. As it slides down the vertical wall, it engulfs the smaller drops in its path, growing in size and velocity with each coalescence. Coalescence occurs rapidly due to the need to correct large curvatures in the film-free surface and to minimize surface energy. This simulation shows how readily coalescence will occur when two volumes of fluid come into contact with each other. It also demonstrates the model's ability to use contact angle information from the disjoining pressure term to recover a circular droplet footprint quickly after a perturbation to the contact line. Finally, the simulation qualitatively reproduces, albeit on a smaller scale, the coalescence events present in figure 1*a* and its electronic supplementary material.

In relation to the observations in figure 1*b*, figure 4 depicts two simulations of coalescence events. In this case, we consider a horizontal plane substrate so that our analysis is not confounded by sliding motions of the droplets. Figure 1*b* indicates a very small contact angle, so we have set *θ*_e_=1° for the first simulation. The initial condition (*t*=0) involves seven droplets placed in no particular pattern in a square domain. Each has been assigned an initial height of *h*_0_=2 and radius $r_{0}=\frac{1}{2}$ to encourage spreading.

As soon as the simulation begins, the drops spread outwards and decrease in height in order to reach an equilibrium position. The meeting of contact lines between neighbouring drops leads to coalescence of the seven drops into one single body of fluid by *t*=0.5, which quickly recovers the circular footprint shown at *t*=10. The end result is a single drop sitting at rest on the horizontal plane with roughly equal radius and height. Our simulations indicate that the same qualitative outcome occurs for any non-zero value of *θ*_e_. However, the actual spread area differs greatly with *θ*_e_; a smaller contact angle means a larger *R* scale and smaller *H*, so a drop with *θ*_e_=1° is considerably more spread out in dimensional space at equilibrium than a drop with *θ*_e_=50°.

The same simulation was then run for the special case *θ*_e_=0°. Setting the contact angle to zero removes the disjoining pressure term from the model and allows the fluid to be completely wetting. This is clear at times *t*=0.5 and *t*=10, when the drops coalesce and then continue to spread rather than forming a circular footprint. While we have halted the simulation at *t*=10, the spreading will continue at a slow pace for all time. A choice of *θ*_e_=0° appears to be most representative of the coalescence behaviour observed in figure 1*b*.

Coalescence is the key mechanism behind spray coverage on leaves, but the reverse process of separation may sometimes occur too. It is well known that pearling occurs at the rear of a drop travelling over a surface at a sufficient velocity [20,21]. However, we are presently more interested in the mechanism of droplet break-up due to heterogeneities in the surface. The wetting properties of a leaf are determined by the surface microstructure, and the integrity of this structure can be compromised by small physical and chemical interferences. The microstructure may also vary along the topography of the leaf, particularly between the cuticle and veins.

**Figure 5.**
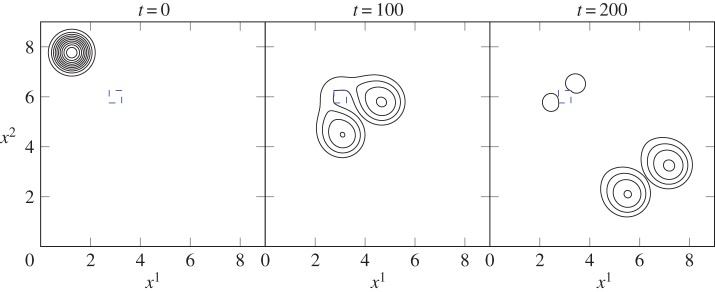
As a drop flows down an inclined plane with equilibrium contact angle *θ*_e_=15°, it encounters a small surface defect with *θ*_e_=45° (blue dashed square). This causes separation into four smaller droplets as the defect obstructs the path of movement. Parameter values are *γ*=0.072 N m^−1^, *ϵ*=0.13 and Bo=0.84, contour spacing is Δ*h*=0.07.

Figure 5 considers the case in which there is a small heterogeneity present in the path of a sliding drop, leading to a change in contact angle on the surface. The substrate is an inclined plane with the highest point at the top left corner of the domain, and lowest at the bottom right. The majority of the surface has a contact angle of *θ*_e_=15° associated with it, and the square defect is more difficult to wet with *θ*_e_=45°. The simulation shows that the drop avoids wetting the defect in favour of the more wettable surface surrounding it and breaks up into four separate droplets: two which continue to flow down the plane, and two of much smaller mass which break off in a process similar to pearling.

The coalescence and separation simulations depicted in figures 3–5 have been presented for comparison with real observations like those in figure 1. Similar droplet behaviours have been studied before [16,18,19,24,29,32,34], but have rarely been studied in the context of spray droplets on leaves.

### 6.2 Droplet movement on a virtual leaf surface

While planar substrates appear to be sufficient for demonstrating simple coalescence processes like those in figure 1, the topographical features of a leaf surface cannot always be ignored. In this section, we investigate the relative roles that gravity and curvature play in droplet motion, using the section of the reconstructed cotton leaf shown in figure 2*b*. This particular leaf section was chosen so that there were interesting topographical features present to influence the flow.

**Figure 6.**
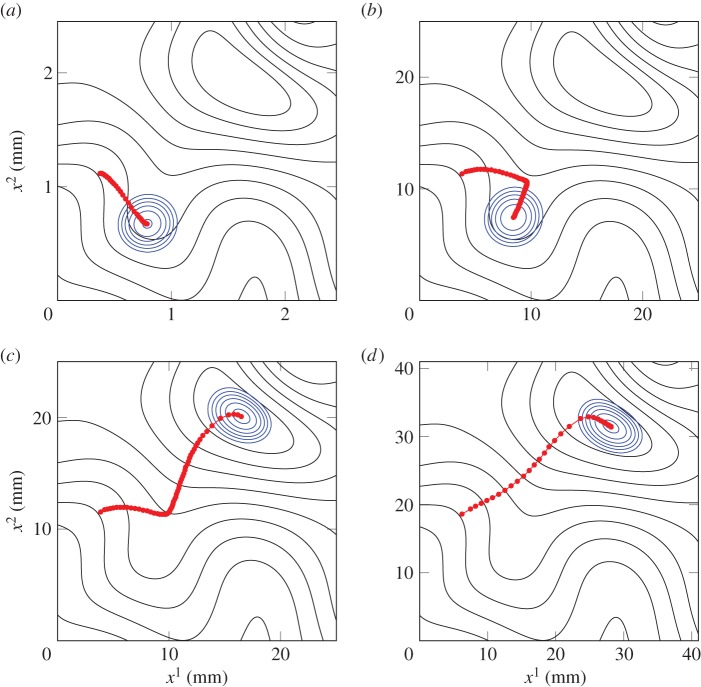
A (*a*) 0.014 μl, (*b*) 14 μl, (*c*) 16 μl and (*d*) 65 μl drop has been placed on the virtual cotton leaf surface (black contours), and its movement simulated for a time of *t*=40 (dimensionless) units. The blue contours represent the final position of the droplet, while the red dotted path illustrates the path it took to arrive there (at intervals of Δ*t*= 2/3). The contour spacing is 0.1*H*_s_ mm for the leaf topography and 0.2*H* mm for the drops. See also the electronic supplementary material.

Consider figure 6*a* (and electronic supplementary material). Here, a drop of representative spray diameter 0.3 mm has been placed on the leaf section of figure 2*b*, and the simulation allowed to evolve in time. The contact angle for this simulation (and all following) is *θ*_e_=50°, leading to a scaling of *R*=0.249 mm and *H*=0.109 mm. Figure 6*a* is shown in dimensional variables (in units of mm) for ease of interpretation. The droplet (blue contours) is shown is its *final* position, while the (red) dotted line illustrates the path it took to arrive there. The drop was initially placed near a high point (a ‘peak’) of the leaf surface, and by the end of the simulation it has not travelled far from this point. The path indicates that the drop moved down the side of the peak and nestled itself in a trough between the initial peak and a neighbouring one. Here, the drop is at rest and ceases to move.

This path of movement is contrary to the path one might imagine the drop to take if driven by gravity, but it is not surprising given the small droplet size. After all, a drop of diameter 0.3 mm has volume of only 0.014 μl. As the mass and consequent gravitational influence on the drop is small, substrate curvature acts as the primary driving force of motion. Gradients in substrate curvature tend to direct the drop to a region of the substrate where the surface energy of the fluid can be minimized.

Figure 6*b*–*d* (and the electronic supplementary material) shows similar simulations, but with drops of varying size. Drops of 14, 16 and 65 μl are considered, respectively (corresponding diameters, volumes, Bond numbers and length and height scales are shown in table 1), which are generally outside the realm of possible droplet volumes produced from an agricultural spray nozzle, but serve to provide examples of the effect that increased mass has on droplet movement. In order to obtain a fair comparison between the simulations, the leaf surface has been scaled up in size in order to retain identical topographical features in relation to the size of the drops. This leads to a scale factor for the length and height scales of each simulation when compared with the reference simulation of figure 6*a*. We define these scale factors as *H*_s_=*H*/*H*_300_, where *H*_300_=0.109 is the height scale for a standard 0.014 μl drop. Note that *L*_s_ has the same value as *H*_s_ for these simulations, because the aspect ratio of the drops does not change. The scale factor values are listed in table 1 and are evident in the differently sized domains in figure 6.

**Table 1.** Parameters used in the simulations depicted in figures 6 and 7.

*D* (mm)	*R* (mm)	*H* (mm)	*V* (μL)	Bo	*H*_s_,*L*_s_	40*T* (s)
0.3	0.249	0.109	0.014	0.0085	1	0.0044
3.0	2.492	1.087	14	0.8451	10	0.0445
3.1	2.575	1.123	16	0.9023	10 $\frac{1}{3}$	0.0460
5.0	4.153	1.812	65	2.3474	16 $\frac{2}{3}$	0.0742

figure 6*b* shows the path taken by a drop initially placed in the same position as the one in figure 6*a* (this is true for all of the simulations in this figure). Rather than immediately moving towards the valley, the 14 μl drop begins to slide down the peak somewhat before making a sharp turn. The drop then moves a short way uphill to arrive in the valley like the first drop. Figure 6*c* sees a drop of similar volume (16 μl) move along the topography. This drop initially has a very similar path, but ultimately veers in the opposite direction when it makes a left turn. The drop comes to rest within the deep valley in the upper-right portion of the domain, which happens to be the lowest point of the given topography. Finally, in figure 6*d*, we see a very large drop of 65 μl. This drop appears to take the path of steepest descent along the topography, and very quickly moves towards the same deep valley. The drop becomes ‘caught’ in this topographical feature and cannot move any further.

These simulations show a gradual change in the balance between substrate curvature gradients and gravity effects on the flow, in response to a change in droplet mass. The smallest drop, in figure 6*a*, is driven purely by gradients of curvature in the the leaf surface. Figure 6*b*,*c* shows two drops of very similar size being influenced by a combination of the two driving forces. We see that substrate curvature gradients prevail for figure 6*b*, while the balance is tipped in favour of gravity in figure 6*c*. In figure 6*d*, gravity dominates the flow and substrate curvature appears to have little measurable effect.

It is not surprising to find that typical spray-size droplets do not move in response to gravity. Indeed, many small stationary drops can be seen in figure 6*a*, and movement is not observed until sufficient coalescence has occurred. The nature of an agricultural spray means that drops do usually land on the leaf in close proximity to each other and are often even impacted from above by other spray drops.

**Figure 7.**
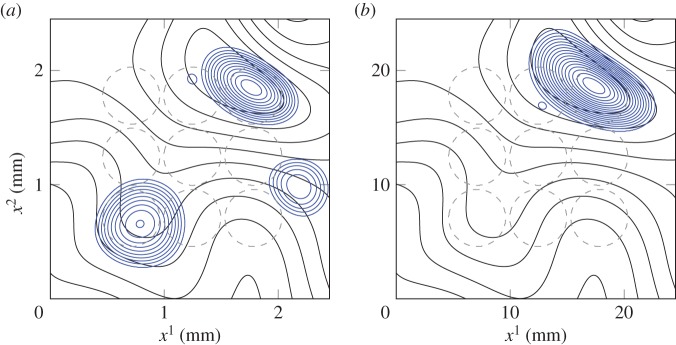
Nine (*a*) 0.014 μl and (*b*) 14 μl drops are placed on the virtual cotton leaf surface (black contours), and their movement simulated for a time of *t*=40 (dimensionless) units. The blue contours represent the final positions of the droplets, while the grey dashed contours represent their initial positions. The contour spacing is 0.1*H*_s_ mm for the leaf topography and 0.2*H* mm for the drops. See also the electronic supplementary material.

In figure 7*a*,*b* (and electronic supplementary material), we have placed a grid of nine equally spaced 0.014 μl and 14 μl drops (respectively) on the leaf surface in order to represent the adhesion of multiple spray drops. At the beginning of the simulation, the drops start to move in different directions according to substrate curvature gradients and gravity. In figure 7*a*, the nine drops coalesce to form three larger drops. It appears that even with this combined mass, the drops are still small enough to be driven primarily by substrate curvature gradients, as they have not all moved downhill. Contrastingly, the larger drops in figure 7*b* have coalesced to form one single drop which has settled in the lowest part of the leaf. The supporting animation for these two simulations shows the coalescence between droplets in much more detail.

While these simulations have evolved for *t*=40 units of dimensionless time, the real time scales (40 *T*, where *T*=3*μR*^4^/(*γH*^3^)) are listed in table 1. The times range from 0.0044 to 0.0742 s, indicating that the droplet movement is very fast. It is known that the presence of a precursor layer can create a disparity between real and simulation time scales [16,19,24], as the precursor film removes the true contact line and thus allows the main body of fluid to move more easily. The enhanced movement can only be counteracted by reducing the precursor thickness, and consequently the mesh spacing, which can be computationally prohibitive.

## 7. Discussion

In this study, we have investigated droplet motion in response to various physical and chemical properties of the surface. Our simulations replicate coalescence events that were observed experimentally for two contrasting spray scenarios (figure 1) and are more generally observed when spraying leaves. First, a drop sliding down a vertical wall engulfed smaller droplets in its path, and then drops spreading on a horizontal surface with a small (or zero) equilibrium contact angle formed a thin coating film. By assigning a spatially varying contact angle to the surface, separation events were also simulated when drops preferentially wetted areas with a smaller contact angle.

Of central interest to the study was the interplay that occurred between gravity and substrate curvature effects for drops on a complexly curved virtual leaf surface. A surface reconstructed from scans of a cotton leaf was used as a realistic topography for our droplet motion. This surface, coupled with a thin film model defined in a curvilinear coordinate system [44–47], allowed for realistic simulations of the post-impaction behaviour of spray droplets. We observe that a typical-sized spray droplet from a medium spray [53] was primarily driven by gradients in the curvature of the leaf surface, and that a much larger droplet mass would be required for gravity to have a significant effect on the flow. However, in the context of an agricultural spray, there is usually a larger number of drops present on the leaf, allowing the opportunity for drops of increased mass to form through coalescence. This is when runoff occurs. For drops of any size, our simulations showed that certain features of the leaf topography could ‘catch’ the drops and inhibit any further movement. The results illustrate that there is an important interplay between substrate curvature gradients and gravity present for drop movement on a complexly curved substrate, which cannot be ignored in the governing model.

The focus of this study has been to implement simulations of droplet movement on a realistic leaf surface topography, focusing particularly on the effect of substrate curvature. Coalescence between drops was also simulated, and we postulated that defects in the leaf surface, resulting in a heterogeneous contact angle, could act as an obstruction and cause drop separation. However, real leaf surfaces are very complex in nature; accurate description of the surface requires knowledge of its microstructure, and the position of anatomical features such as veins and stomata. Surface microstructure may vary significantly over the single leaf surface; for example, leaf veins often have different properties from the rest of the leaf surface. Further, hairs may be present in dense or sparse patterns, altering the topography or acting as obstacles to droplet movement. The spray formulation itself contains surfactants, which dynamically change the surface tension of the fluid. Evaporation of water from the formulation causes the surfactant concentration to change over time, in addition to the volume and mass of the drop. The current thin film framework does not strictly apply for non-small contact angles and is certainly not valid for angles greater than 90°, which may present a problem for particularly hydrophobic species. While some of these complications have received attention before [25,32], the topic of post-impaction modelling in the context of agricultural sprays certainly deserves further attention to improve realism.

## Supplementary Material

Videoforfigure1a.mp4 Video for figure 1a The abaxial surface (underside) of an avocado leaf is sprayed with water, leading to coalescence and runoff. Videoforfigure1b.mp4 Video for figure 1b The abaxial surface (underside) of an avocado leaf is sprayed with a surfactant mixture, causing a coating film to form on the leaf surface. Videoforfigure6a.mp4 Video for figure 6a A 0.014 microlitre drop is placed on a virtual cotton leaf surface. The drop moves in response to substrate curvature gradients. Videoforfigure6b.mp4 Video for figure 6b A 14 microlitre drop is placed on a virtual cotton leaf surface. The drop moves in response to substrate curvature gradients and gravity. Videoforfigure6c.mp4 Video for figure 6c A 16 microlitre drop is placed on a virtual cotton leaf surface. The drop moves in response to substrate curvature gradients and gravity. Videoforfigure6d.mp4 Video for figure 6d A 65 microlitre drop is placed on a virtual cotton leaf surface. The drop moves in response to gravity. Videoforfigure7a.mp4 Video for figure 7a Nine 0.014 microlitre drops are placed on a virtual cotton leaf surface. The drops move in response to substrate curvature gradients, and coalesce to form larger drops. Videoforfigure7b.mp4 Video for figure 7b Nine 14 microlitre drops are placed on a virtual cotton leaf surface. The drops move in response to substrate curvature gradients and gravity, and coalesce to form a single large drop.

## Appendix A. Finite volume discretization

If we return to the coordinate-free form of (3.16), we have

A 1 $$\partial_{t}h=-\nabla \cdot \text{J}.$$

We discretize this with a vertex-centred finite volume scheme. For simplicity, node spacings in the *x*^1^ and *x*^2^ directions are kept constant, though Δ*x*^1^ and Δ*x*^2^ may be different from one another. Integrating (A 1) over the area of a CV at a point $P=(x_{P}^{1},x_{P}^{2})$ on the curved substrate, where

A 2 $$\left.\begin{array}{rl} x_{w}^{1} & =x_{P}^{1}-\frac{1}{2}\Delta x^{1}\leq x^{1}\leq x_{P}^{1}+\frac{1}{2}\Delta x^{1}=x_{e}^{1}\\ \;\text{and}\;x_{s}^{2} & =x_{P}^{2}-\frac{1}{2}\Delta x^{2}\leq x^{2}\leq x_{P}^{2}+\frac{1}{2}\Delta x^{2}=x_{n}^{2}, \end{array}\right\}$$

defines the limits of the CV *V*_*P*_, we have

A 3 $$\begin{array}{rl} \iint_{V_{P}}\partial_{t}h\;dV & =-\iint_{V_{P}}\nabla \cdot \text{J}\;dV\\ & =-\oint_{\partial V_{P}}\text{J}\cdot \hat{\text{n}}\;dS\\ & =-\int_{e}\text{J}\cdot \text{i}\;dS+\int_{w}\text{J}\cdot \text{i}\;dS-\int_{n}\text{J}\cdot \text{j}\;dS+\int_{s}\text{J}\cdot \text{j}\;dS. \end{array}$$

The second line is a result of the divergence theorem, e, w, n, s indicate the east, west, north and south faces of the CV, and **i, j** are the regular Cartesian basis vectors. A midpoint approximation of (A 3) gives

A 4 $$A_{P}\partial_{t}h_{P}=-\Delta x_{e}^{2}J_{e}^{1}+\Delta x_{w}^{2}J_{w}^{1}-\Delta x_{n}^{1}J_{n}^{2}+\Delta x_{s}^{1}J_{s}^{2},$$

where *A*_*P*_ is the surface area of the substrate within the CV at point *P*, $\Delta x_{e}^{2}$ is the length of the east CV face along the curved substrate, $J_{e}^{1}=J^{1}(h(x_{e}^{1},x_{P}^{2}))$ indicates evaluation at the centre of the east CV face, and similarly for the remaining terms. The area and length measurements of the curved substrate may be calculated directly, or approximated with the metric tensor components (for example, $\Delta x_{e}^{2}\;\approx G_{22}\Delta x^{2}$ for sufficiently small Δ*x*^2^). Witelski & Bowen's [54] *pL*2 method approximates the time derivative of (A 4) with the second-order backward differentiation formula

A 5 $$\partial_{t}h_{P}{|}_{t^{n+1}}\approx \frac{3h_{P}^{n+1}-4h_{P}^{n}+h_{P}^{n-1}}{2\Delta t}.$$

The flux **J** (see (3.17)) involves a number of parameters which vary over the leaf topography; at each node, there is a unique gravitational force vector, metric tensor and curvature tensor. Equation (A 4) indicates that these parameters (and in many cases, their derivatives) must be known at each CV face.
